# Visual impairment and medication safety: a protocol for a scoping review

**DOI:** 10.1186/s13643-021-01800-8

**Published:** 2021-09-15

**Authors:** Sally J. Giles, Maria Panagioti, Lisa Riste, Sudeh Cheraghi-Sohi, Penny Lewis, Isabel Adeyemi, Karen Davies, Rebecca Morris, Denham Phipps, Christine Dickenson, Darren Ashcroft, Caroline Sanders

**Affiliations:** 1 NIHR Greater Manchester Patient Safety Translational Research Centre, University of Manchester, Suite 11, 7th floor, Williamson Building, Oxford Road M13 9PL, Manchester, UK; 2 Centre for Primary Care, University of Manchester, Oxford Road M13 9PL, Manchester, UK; 3 Division of Pharmacy and Optometry, School of Health Sciences, Faculty of Biology, Medicine and Health, University of Manchester, Manchester Academic Health Sciences Centre (MAHSC), Manchester, UK

**Keywords:** Medication safety, Visual impairment

## Abstract

**Background:**

The number of individuals with a visual impairment in the UK was estimated a few years ago to be around 1.8 million. People can be visually impaired from birth, childhood, early adulthood or later in life. Those with visual impairment are subject to health inequities and increased risk for patient safety incidents in comparison to the general population. They are also known to be at an increased risk of experiencing medication errors compared to those without visual impairment. In view of this, this review aims to understand the issues of medication safety for VI people.

**Methods/design:**

Four electronic bibliographic databases will be searched: MEDLINE, Embase, PsycInfo and CINAHL. Our search strategy will include search combinations of two key blocks of terms. Studies will not be excluded based on design. Included studies will be empirical studies. They will include studies that relate to both medication safety and visual impairment. Two reviewers (SG and LR) will screen all the titles and abstracts. SG, LR, RM, SCS and PL will perform study selection and data extraction using standard forms. Disagreements will be resolved through discussion or third party adjudication. Data to be collected will include study characteristics (year, objective, research method, setting, country), participant characteristics (number, age, gender, diagnoses), medication safety incident type and characteristics.

**Discussion:**

The review will summarise the literature relating to medication safety and visual impairment.

## Background

Visual impairment (VI) refers to a condition where the person has a reduction in their visual acuity or field of vision, which cannot be corrected by spectacles or contact lenses. It is estimated to affect 285 million people, 39 million of whom are blind [1]. The number of individuals with a visual impairment in the UK was estimated a few years ago to be around 1.93 million [2]: about 3% of the adult population [3]. People can be visually impaired from birth, childhood, early adulthood or later in life. People over 50 represent the largest group of blind and visually impaired people, which currently stands at 82% of the blind and visually impaired population worldwide [1]. Those with visual impairment or blindness are reported to be marginalised in terms of accessing healthcare information and facilities, and they are known to receive sub-optimal healthcare [4]. Marginalised patients, such as those with visual impairment, are subject to health inequities and increased risk for patient safety incidents in comparison to the general population [5]. Studies also suggest that people with VI are more at risk of social isolation [6], which could further perpetuate their increased risk of being affected by patient safety incidents. Those with visual impairment are known to be at an increased risk of experiencing medication errors [7] compared to those without visual impairment. This could be for a number of reasons, such as being unable to open medication containers, being unable to differentiate various types of medication containers, being unable to differentiate various types of tablets/capsule dosage forms, forgetting to take medication on time and taking the wrong medication [7, 8]. Medicine-taking is complex and requires various and coordinated forms of work on the part of the patient and those in their networks. People with VI may not be able to perform their medicine work as easily as those without VI. The importance of supporting visually impaired patients to help improve their medication safety was further strengthened in 2016 when NHS England introduced the Accessible Information Standard (AIS), which required that the information and communication needs of disabled patients are proactively addressed in all aspects of NHS healthcare, including the dispensing of medicines [9]. Digital solutions are increasingly used to improve people’s safety and quality of life and VI people are frequent users of the Internet to support their wellbeing [10, 11]. There are concerns about the cost and lack of universal availability of this technology [12], and it is less clear how digital technologies, managed by individuals, can not only empower visually impaired people, but also safeguard them from harm related to medication management [13].

## Rationale

To understand the issues of medication safety for VI people, it is essential to gather evidence from their experience and consider that alongside previous research. We have undertaken focus groups with VI people identifying a number of key themes, including the variation in VI and consequences for medication safety. We propose conducting a scoping review of existing research evidence to increase the understanding of VI and medication safety.

The issues of medication safety for people with VI depend on the nature/severity of the visual condition. Previous research conducted by the team [14] identified that health and social care practitioners lack knowledge and understanding of VI, but many people with VI feel confident to explain what works for them and find “work arounds” to maintain safety in medicine taking.

With an ageing population in many developing countries, the number of people with age-related vision loss is likely to increase. The scoping review aims to identify the specific medication safety issues for people with visual impairment and how these patients manage their medicines to mitigate against medication safety issues.

### Objective of the scoping review

To identify and analyse the existing literature relating to the association between medication safety and visual impairment, including the main types of and contributory factors to medication safety issues.

## Methods

Firstly, “to examine the extent, range and nature of research activity; this type of rapid review might not describe research findings in any detail but is a useful way of mapping fields of study where it is difficult to visualise the range of material that might be available.” The second common reason is determining whether a systematic review is feasible and of value. The third and fourth common reasons seem to describe scoping reviews that are not exploratory or preparatory, but done in their own right. Other authors similarly state that scoping reviews are used to synthesise research evidence and are often used to map existing literature in a given field in terms of its nature, features and volume. As such, scoping reviews have also been called “mapping” reviews. Finally, a map of the range of the available evidence can be undertaken as a preliminary exercise prior to the conduct of a systematic review. If we wish to conduct other more systematic reviews, then this review will allow us to see where and if that might be possible.

### Search strategy

Four electronic bibliographic databases will be searched: MEDLINE, Embase, PsycInfo and CINAHL. We will also identify eligible studies by checking the reference lists of those studies identified in the search that meet our inclusion criteria. Our search strategy will include search combinations of two key blocks of terms: visual impairment and medication safety (Table 1), similar to those used in two previous reviews [15, 16].

**Table 1:** Main search terms

Search terms	Search block	Source
1 (preventable adverse drug or medication related adverse event?).ti. 2 Medication errors/ or Inappropriate Prescribing/ 3 (medication safety or medication incident? or medication error?).ti,ab. 4 ((pharmacist? or prescrib$ or prescription? or dispens$ or dosing) adj2 (error? or mistake? or miscalculat$)).ti,ab. 5 (medication? adj2 misadventure?).ti,ab. 6 ((inappropriate adj3 (prescription? or medication?)) or ((appropriat$ or inappropriat$ or optimal) adj2 prescrib$)).ti,ab. 7 effective prescribing practice?.ti,ab. 8 medication reconciliation/ [...done to avoid medication errors.] 9 (quality improv$ and (prescrib$ or prescript$ or dosing)).ti. 10 ((weight-based or surface-based or weight independent) adj2 (prescrib$ or dose or dosing or dosage?)).ti,ab. and (safety or error?).ti,hw. 11 ((drug? or medication? or medicine? or dose or dosage? or dosing) adj2 wrong$).ti,ab. 12 (medication? adj2 (reconciliation? or audit? or quality improvement)).ti. 13 (accident$ adj2 overdose?).ti,ab. 14 (near miss or near misses).ti,ab. 15 ((excess$ or inadequat$) adj2 (dosage? or dose? or dosing)).ti,ab. 16 (“medication related” adj2 (problem? or issue? or hospitali?ation? or mortal$ or morbid$ or illness$ or condition?)).ti,ab. 17 Medical Order Entry Systems/ and (prescript$ or prescrib$).ti,hw. 18 Decision Support Systems, Clinical/ and (prescrib*.ti,hw. or medication?.ti. or *drug therapy/) 19 “Drug Therapy, Computer-Assisted”/ and (safety or error?).ti. 20 Electronic prescribing/ and (safety or error? or improv$).ti. 21 (prevent$ and (error? or (adverse adj2 event?))).ti. and (dosing or drug? or medication? or prescript$ or prescrib$).ti,hw. 22 ((drug? or medication? or prescrib$) adj3 error?).ti. and ((prevent$ or reduce? or reducing).ti. or pc.fs.) 23 “Pharmaceutical Preparations”/ae and (prevent$.ti. or (prevention or preventing).hw.) 24 (Pharmaceutical preparations/ or Drug Therapy/ or exp Drug Administration Routes/ or exp Drug administration schedule/ or exp drug delivery systems/ or drug dosage calculations/ or exp drug prescriptions/ or exp drug therapy, Combination/ or Drug Therapy, Computer-assisted/) and (error? or mistake or mistakes or prevent$ adverse).ti. 25 (Medication Systems, Hospital/ or Pharmacy Service, Hospital/) and ((error? or mistake or mistakes or prevent$ adverse).ti. or ((prevent$ or reduce? or reducing) adj2 (error? or adverse event? or adverse drug event? or medication related problem?)).ab.) 26 (exp therapeutic uses/ or exp anti-infective agents/ or exp anti-bacterial agents/) and (((prevent$ or reduce? or reducing) and (error? or (adverse$ adj3 event?))) or (inappropriat$ adj2 “use”)).ti. 27 Medical errors/pc and (medication? or drug?).ti,ab. 28 (exp therapeutic uses/ or exp anti-infective agents/ or exp anti-bacterial agents/) and Medical Errors/ 29 (Pharmaceutical preparations/ or Drug Therapy/ or exp Drug Administration Routes/ or exp Drug administration schedule/ or exp drug delivery systems/ or drug dosage calculations/ or exp drug prescriptions/ or exp drug therapy, Combination/ or Drug Therapy, Computer-assisted/) and Medical Errors/ 30 (prevent$ adverse drug or (causes adj2 (prescri$ error? or medication? error?)) or medication related adverse).ti,ab. 31 Medication errors/pc or Inappropriate Prescribing/pc 32 or/2-31 [Med Errors]	Medication safety	See Maaskant et al, 2015
exp Visually Impaired Persons/ or (Visually adj3 Impaired adj3 Person*).tw. 2) exp Vision Disorders/ 3) exp Blindness/ or blindness.tw. 4) exp Vision, Low/ 5) ((low* or handicap* or subnormal* or impair* or partial* or disab* or reduce* or diminish* or decrease* or problem* or disorder* or loss or disease* or defect* or disturb*) adj3 (vision or visual* or sight* or eye or eyes or eyesight or sight)).tw. 6) Blind*.tw. 7) (rehabilitat* adj4 (vision or visual* or sight* or eye or eyes or sight or eyesight)).tw.	Visual Impairment	Taken from Kentab, 2019

The proposed search terms are shown in Table 1.

### Eligibility criteria

Studies will be excluded if they fail to meet any of the 3 criteria (a “NO” choice). Studies will be eligible for full-text screening if they fully (a “YES” choice to each criterion) or partly (one or more “UNSURE” choice) meet criteria A1, A2 and A3.

A. For any study type (including review articles and opinion pieces):

1. Is it an empirical research?
  YES, NO, UNSURE

2) Does it make reference to medication safety or medicines management?

YES, NO, UNSURE

3) The research has been conducted with visually impaired patients?
  YES, NO, UNSURE

We will include:

*Types of studies*: We will include empirical studies which provide data on medication safety or medicines management for visually impaired patients. Study designs will not be restricted and will include both quantitative designs (that is, randomised controlled trials, quasi-experimental studies, cohort studies, cross-sectional studies) and qualitative studies including case studies. We will also include grey literature reports.

*Types of participants:* patients with visual impairment. We will not exclude participants on the basis of comorbidities

*Phenomena of interest:* medication safety/medicines management issues for visually impaired patients. On the basis of previous research, we anticipate that such issues may include being unable to open medication containers, being unable to differentiate various types of medication containers, being unable to differentiate various types of tablets/capsule dosage forms, forgetting to take medication on time and taking the wrong medication and polypharmacy (Zhi-Han, 2017: Cheraghi-Sohi et al, 2014.

*Setting/context:* Studies conducted in any setting. We will not restrict our search in specific geographical areas or date of publication.

We will exclude:

- Non-empirical studies
- Case studies reporting a new onset of visual impairment following medication use
- Articles in non-English languages

### Management of search outcomes and study eligibility screening

The results of the searches of each database will be exported to COVIDENCE [1] and duplicates deleted.

**Fig. 1:**
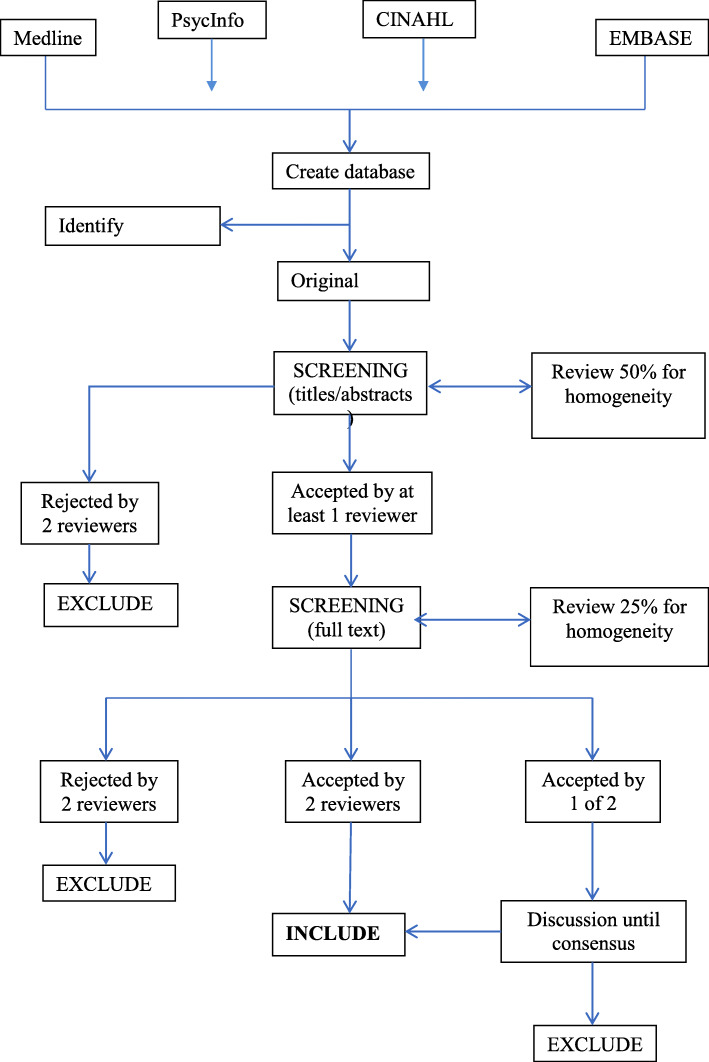
PRISMA flowchart

Using PRISMA guidelines (Moher et al, 2009), screening will be completed in two stages (see Fig. 1). Initially, the titles and abstracts of the identified studies will be screened for eligibility (see the “Eligibility criteria” section). A proportion of titles and abstracts (50%) will be screened by two researchers independently to assess reliability using the kappa statistic. Assuming reliability is confirmed, screening of the remaining titles and abstracts will be completed by one reviewer.

Next, the full texts of studies initially assessed as “relevant” for the review will be retrieved and checked against our inclusion/exclusion criteria. Full-text screening will be completed by two members of the research team independently, with disagreements resolved by discussion.

### Methodological quality of the studies

As scoping reviews aim to provide a map of what evidence has been produced as opposed to seeking only the best available evidence to answer a particular question related to policy and practice, a formal assessment of methodological quality of the included studies of a scoping review will not be performed.

### Data synthesis

A narrative synthesis will be conducted and the results will be organised according to the research aims. The first section of the results will present the research findings on the association between medication safety and visual impairment, the main types of patient safety issues encountered by people with visual impairment and the key contributory factors. The second section will focus on presenting the available evidence on the use of digital technologies for managing medication among people with visual impairment. In the third section, we will outline future research recommendations for designing and testing digital interventions to improve medication safety in people with visual impairment. We will take into consideration the present findings as well as the broader literature (from existing systematic reviews) on the use of digital technologies in improving medication safety.

## Discussion

This review will summarise the literature relating to visual impairment and medication safety. Four electronic bibliographic databases will be searched, using combinations of two key blocks of search terms: visual impairment and medication safety. The findings from this review will provide an evidence base for further work in this area. It will increase understanding of the issues that visually impaired people face in relation to medication safety and ultimately improve quality of health care.

## Data Availability

Not applicable

## Abbreviations

VI: Visual impairment
SG: Sally Giles
LR: Lisa Riste
RM: Rebecca Morris
SCS: Sudeh Cheraghi-Sohi
PL: Penny Lewis
AIS: Accessible Information Standard
